# Clinicopathological features and prognostic analysis of 30 patients with laryngeal and hypopharyngeal adenoid cystic carcinoma: a single-center retrospective study

**DOI:** 10.1007/s00432-026-06449-1

**Published:** 2026-04-08

**Authors:** Mingzhu Wang, Jing Zhou, Tingyao Ma, Xiaohong Chen

**Affiliations:** Department of Otolaryngology-Head and Neck Surgery Beijing Tongren Hospital, Capital Medical University, DongJiaoMinXiang Street, DongCheng District, Beijing, 100730 People’s Republic of China

**Keywords:** Laryngeal and hypopharyngeal tumors, Adenoid cystic carcinoma, Pathological characteristics, Prognosis

## Abstract

**Purpose:**

To evaluate clinical characteristics, treatment outcomes, and prognostic factors in patients with laryngeal and hypopharyngeal adenoid cystic carcinoma (LHACC).

**Methods:**

This retrospective, single-institution study (2013–2023) included patients with pathologically confirmed LHACC who underwent primary surgical treatment. Clinical and pathological variables were reviewed, and relevant gene alterations were extracted from cBioPortal. Overall survival (OS) and disease-free survival (DFS) were estimated using Kaplan–Meier curves and compared by the log-rank test.

**Results:**

Thirty patients were analyzed (median follow-up, 36 months; range, 4–130). The cohort comprised 10 men and 20 women (mean age 51 years). Hoarseness (36.7%) was the most frequent symptom and correlated with poorer OS (*P* = 0.033). Locoregional recurrence occurred in 5 patients (16.7%), and distant metastases in 15 (50%). OS rates at 3, 5, and 10 years were 95.5%, 76.4%, and 38.2%; DFS rates were 79.2%, 49.4%, and 10.3%. Survival rates were estimated using the Kaplan–Meier method, with 9 censored patients who had follow-up shorter than 3 or 5 years without tumor progression or death. Higher Ki-67 index (> 15%) predicted an unfavorable DFS (*P* = 0.041). Gene fusions (4 MYB–NFIB, 1 CTNNA3–NFIB) were detected in 50% of tested cases, highlighting molecular features of LHACC.

**Conclusions:**

Hoarseness and Ki-67 may help identify high-risk LHACC patients. Surgical resection remains the cornerstone of therapy, but individualized management and long-term follow-up are essential. Frequent MYB–NFIB fusions underscore the molecular basis of LHACC and may inform future prognostic assessment and therapeutic strategies.

**Supplementary Information:**

The online version contains supplementary material available at 10.1007/s00432-026-06449-1.

## Introduction

Adenoid cystic carcinoma (ACC) is a rare head and neck malignancy, typically arising from salivary glands. Although ACC often follows an indolent course, it is characterized by perineural invasion, submucosal extension, and a tendency for late distant metastasis, contributing to poor long-term outcomes (Kokemueller et al. 2004; Ellington et al. 2012; Coca-Pelaz et al. 2015). Laryngeal and hypopharyngeal ACC (LHACC) is extremely rare, accounting for less than 1% of all malignancies in these regions (Naim et al*.*, 2019), whereas squamous cell carcinoma (SCC) remains the predominant histology (Aliabadi et al. 2022). Unlike SCC, which typically exhibits rapid local progression and early nodal involvement (Takes et al. 2012), LHACC generally follows a slow but persistent course.

Patients with LHACC often present with nonspecific symptoms such as hoarseness, dysphagia, or airway obstruction. Such presentations may delay diagnosis and complicate complete surgical resection (Ellington et al. 2012). Histologically, ACC is classified into cribriform, tubular, and solid patterns, with the solid subtype generally associated with poorer prognosis. Most studies on LHACC are limited to case reports or small series (Moukarbel et al. 2008; Cui et al. 2019; Mur et al. 2022), leaving treatment strategies, prognostic factors, and long-term outcomes insufficiently defined. Although reviews have summarized head and neck ACC management in general, few have specifically addressed laryngeal and hypopharyngeal sites, which share anatomical proximity and embryological origin.

Recent studies have identified recurrent genetic alterations in ACC, including MYB–NFIB and CTNNA3–NFIB fusions, as well as deregulation of NOTCH signaling and chromatin remodeling pathways (Shamir et al. 2023; Huang J et al*.*,^2024^). These molecular features may serve as biomarkers for risk stratification and potential therapeutic targets, yet their role in LHACC remains largely unexplored.

To address these gaps, this study aimed to characterize the clinicopathological features of LHACC, evaluate treatment outcomes, and identify prognostic factors, thereby providing evidence to inform individualized management of this rare malignancy.

## Materials and methods

### Study design

This retrospective, single-institution study was conducted at Beijing Tongren Hospital, Capital Medical University, between 2013 and 2023. The study was designed to evaluate the clinical, pathological, and molecular characteristics of laryngeal and hypopharyngeal adenoid cystic carcinoma (LHACC) and to identify prognostic factors associated with survival outcomes. The study was approved by the Ethics Committee of Beijing Tongren Hospital (approval number: TREC2022-KY023), and all participants provided informed consent in accordance with the Declaration of Helsinki.

### Patient selection

Patients were eligible for inclusion if they met all of the following criteria: (1) a histologically confirmed diagnosis of laryngeal adenoid cystic carcinoma (LHACC); (2) receipt of primary surgical treatment involving the larynx; (3) initial diagnosis and/or treatment at our institution, or referral after initial treatment elsewhere with subsequent management and follow-up at our institution; (4) availability of complete clinical, pathological, and follow-up data; (5) provision of written informed consent.

Patients were excluded if they met any of the following criteria: (1) absence of histopathological confirmation of LHACC; (2) incomplete clinical or follow-up data; (3) presence of concurrent malignancies; or (4) loss to follow-up.

### Data collection

Clinical data were retrospectively collected, including patient demographics (age and sex), primary tumor site, presenting symptoms, clinical stage, histopathological features, and treatment modalities. Preoperative evaluation was performed systematically in all patients. The diagnostic work-up included laryngoscope examination, contrast-enhanced CT or MRI of the neck, and chest CT to exclude distant metastasis; PET-CT was conducted when clinically indicated. Treatment decisions were made in a multidisciplinary tumor board in accordance with standard oncologic principles.

Pathological data were extracted from formalin-fixed, paraffin-embedded specimens and independently confirmed by at least two pathologists. Pathological features included histological subtype, tumor grade, high-grade transformation, perineural invasion, vascular invasion, cartilage invasion, surgical margin status, and lymph node involvement. Tumors were graded according to Szanto et al. (1984) based on the proportion of solid components and staged according to the eighth edition of the AJCC TNM system. High-grade transformation (HGT) was defined following Seethala et al., describing conventional adenoid cystic carcinoma that loses its typical cribriform, tubular, or solid architecture and shows poorly differentiated or undifferentiated morphology. Key histologic features include marked cytologic atypia, increased mitotic activity, and necrosis (Ma et al. 2023). All HGT cases in this study met these criteria.

### Molecular data

Publicly accessible genomic data were queried from cBioPortal for Cancer Genomics (www.cbioportal.org). Cases were identified by selecting studies containing adenoid cystic carcinoma and filtering the Primary Site field for “Larynx,” “Hypopharynx,” “Supraglottic,” and “Subglottic” regions. Only cases annotated as adenoid cystic carcinoma in the histology field were included. Extracted molecular data comprised gene fusions, nucleotide and amino acid mutations, mutation types, mutation abundance, and tumor mutation burden. All cases were manually verified to ensure accurate site and histology classification.

### Follow-up and outcome measures

Overall survival (OS) was defined as the time from the initial presentation at our institution to death or last contact. Disease-free survival (DFS) was defined as the time from completion of primary treatment to first locoregional recurrence, distant metastasis, death, or last contact. Locoregional recurrence or distant metastasis was confirmed using imaging and/or pathological evaluation.

### Statistical methods

Statistical analyses were performed using SPSS software (version 27.0; SPSS Inc., Chicago, IL, USA). Associations between tumor location and pathological characteristics were assessed using Fisher’s exact test. OS and DFS were estimated using Kaplan–Meier survival analysis, and survival curves were compared using the log-rank test. Univariate analysis was conducted to identify potential prognostic factors. A two-sided p-value of less than 0.05 (*P* < 0.05) was considered statistically significant.

## Results

### Clinical characteristics

#### Demographics and baseline features

Among the 30 patients with LHACC, 10 were male, and 20 were female. Age at diagnosis ranged from 20 to 76 years (median, 51 years). The primary tumor site was subglottic in 76.7% (23/30), supraglottic in 13.3% (4/30), and post-cricoid in 10.0% (3/30). Hoarseness was the most common symptom (36.7%, 11/30). Symptoms were recorded based on the initial presentation. According to the AJCC 8th edition staging system, 11 patients were classified as stage II, 9 patients as stage III, and 10 patients as stage IV at initial diagnosis. Among the stage IV cases, four patients presented with distant metastases (M1) at diagnosis, all involving the lungs. Two of these patients died at 3 and 4 years after diagnosis.

Specifically, one patient was staged as T4a, two as T3, and one as T2. Kaplan–Meier analysis showed no statistically significant differences in either disease-free survival or overall survival among patients with different clinical stages (all *p* > 0.05). Table 1 summarizes clinical staging, surgical margin status, and other baseline characteristics.

**Table 1:** Basic information of patients with laryngeal and hypopharyngeal adenoid cystic carcinoma and Kaplan–Meier analysis of disease-free survival and overall survival in all patients

Characteristic	n (%)	Disease-free survival			Overall survival		
		3 years (%)	5 years (%)	*P*	3 years (%)	5 years (%)	*P*
Gender							
Male	10 (66.7)	77.3	39.4	0.394	100.0	75.0	0.378
Female	20 (33.3)	83.3	83.3		83.3	83.3	
Age(years)							
≥ 60	4 (3.3)	79.5	51.0	0.867	94.7	80.2	0.339
< 60	26 (83.7)	75.0	37.5		100.0	50.0	
Primary site							
Supraglottic	4 (13.3)	31.3	33.3	0.235	66.7	33.3	0.079
Subglottic	23 (76.7)	85.5	47.4		100.0	83.3	
Post-cricoid area	3 (10.0)	100.0	100		100.0	0.0	
Symptom							
Hoarse	11 (36.7)	87.5	54.7	0.358	100.0	83.3	0.033*
Dyspnea	5 (16.7)	80.0	53.3		100.0	100.0	
Dysphagia	2 (6.7)	100.0	100.0		100.0	100.0	
Pharyngeal parsthesiaane	5 (16.7)	50.0	50.0		50.0	50.0	
Cough and sputum	4 (13.3)	100.0	37.5		100.0	50.0	
Pain	3 (10.0)	50.0	50.0		100.0	100.0	
Clinical stage							
Ⅱ	11 (36.7)	87.5	52.5	0.192	100.0	100.0	0.814
Ⅲ	9 (30.0)	100.0	83.3		100.0	80.0	
Ⅳ	10 (33.3)	57.1	38.1		87.5	65.6	
Margin							
Positive	8 (26.7)	75.0	50.0	0.546	100.0	75.0	0.386
Negative	15 (33.3)	81.0	48.6		93.3	76.4	
Treatment							
Surgery	10 (33.3)	64.8	43.2	0.836	85.7	57.1	0.611
Surgery & Adjuvant therapy	20 (66.7)	86.7	52.5		100.0	83.3	

^*^P < 0.05, **P < 0.01. Values in bold indicate statistically significant differences (P < 0.05).

#### Surgical and adjuvant treatment modalities

Twenty-three patients underwent laryngeal surgery following histological confirmation by direct laryngoscopy or outpatient biopsy: 20 received total laryngectomy, 1 underwent supraglottic partial laryngectomy, and 2 underwent partial laryngectomy. The remaining seven patients did not undergo traditional laryngeal resection; instead, their laryngopharyngeal lesions were excised using transoral laryngeal microsurgery (TLM) and subsequently confirmed as LHACC.

Selective neck dissection was performed during the initial surgery in 11 patients, covering levels II–VI as well as neck and laryngotracheal nodes.

Among the cohort, 10 patients underwent surgery alone, whereas 20 received surgery combined with adjuvant therapy. Postoperative radiotherapy was typically administered in 29–33 fractions for a total dose of 60–66 Gy. Two patients received adjuvant chemotherapy; detailed regimens and reasons for omitting radiotherapy were inconsistently documented. No surgical complications were observed.

All four patients who presented with pulmonary metastases at initial diagnosis underwent tracheotomy along with laryngeal tumor resection or biopsy. None received adjuvant therapy; two of them subsequently underwent total laryngectomy and neck dissection.

#### Survival and tumor progression outcomes

**Fig. 1:**
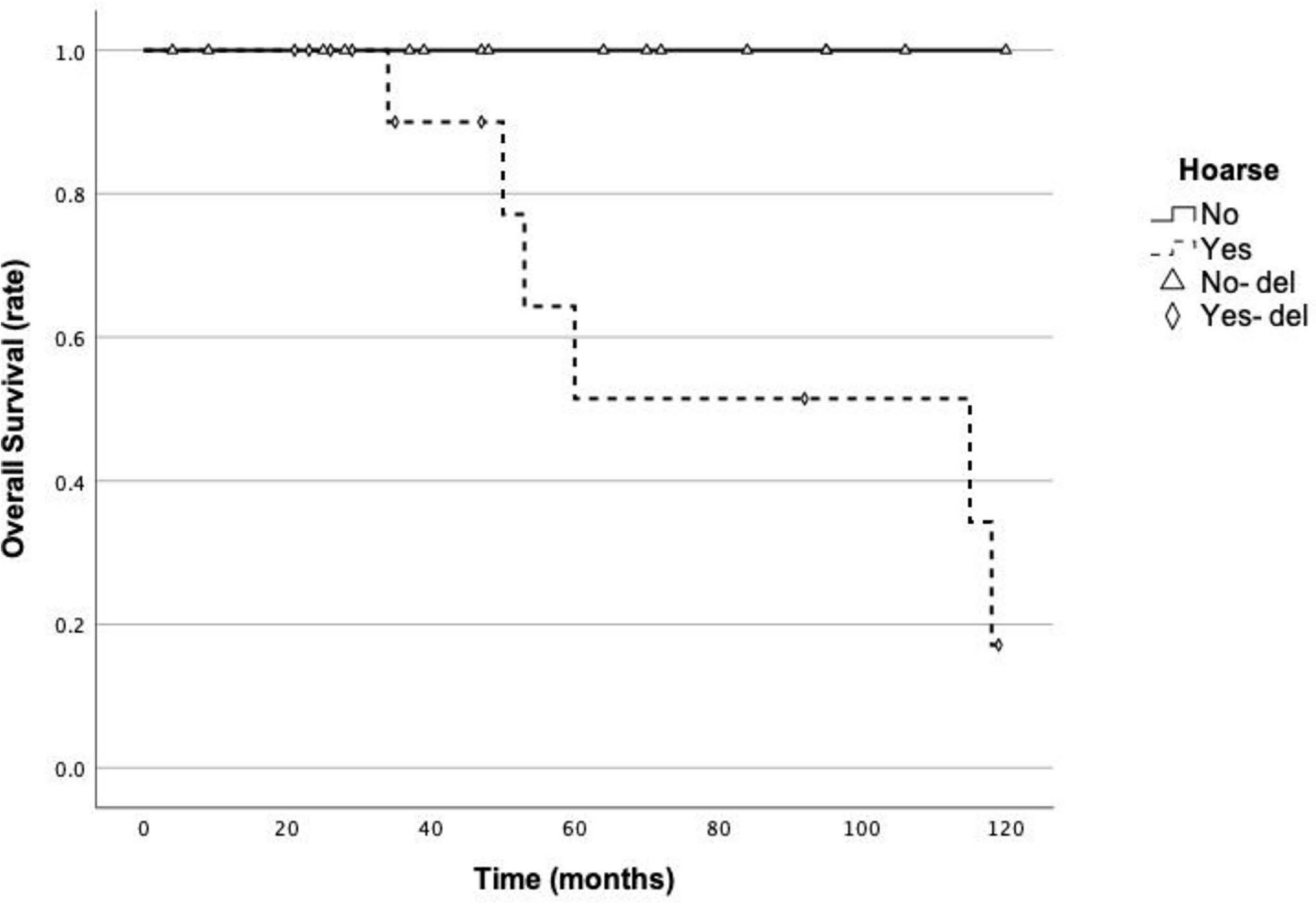
Kaplan–Meier survival analysis stratification chart for hoarse and overall survival (5-year survival rate: 100% vs. 51.4%; log-rank *P* = 0.016)

During a median follow-up of 36 months (range, 4–130), locoregional recurrence occurred in 5 patients (16.7%) at a mean of 60.0 ± 39.99 months. Distant metastases were frequent, with pulmonary involvement in 15 patients (50.0%), including one patient who also developed liver metastasis 15 months after surgery (Online Resource 1). Hoarseness was associated with worse OS (5-year survival: 100% vs. 51.4%; log-rank *P *= 0.016). The Kaplan–Meier curves for stratified OS analyses are shown in Fig. 1. Six patients died during follow-up; survival times among deceased patients ranged from 50 to 118 months (median, 55 months). These outcomes highlight the high rate of late distant metastasis, underscoring the need for long-term surveillance and potential adjunctive systemic or targeted strategies.

### Endoscopic and imaging findings

Laryngoscope findings were available for 22 patients who received initial examination at our institution. Among the 22 evaluable cases, 16 (72.7%) lesions presented as broad-based elevations, and 5 (22.7%) appeared as mildly reddish mucosal masses. The surface was mostly irregular, smooth lesions were rare, and only one case showed a localized hematoma. Detailed laryngoscope features are provided in Online Resource 2.

Among the 16 subglottic tumors, nine involved both the vocal cords and the adjacent subglottic region. Imaging studies, including head and neck MRI, revealed soft tissue masses corresponding to the laryngopharyngeal lesions. For example, one patient exhibited a right vocal cord mass measuring approximately 1.2 cm in diameter, resulting in narrowing of the laryngeal cavity and associated with cough and inspiratory dyspnea (Online Resources 3–7). These endoscopic and imaging features underscore the importance of comprehensive visualization and radiologic assessment in accurately delineating tumor extent.

### Pathological features

#### Local histopathological characteristics

Postoperative pathology confirmed that all 30 patients had LHACC. Based on pathological grading, Grade 1 was the most common, observed in 45.2% (14/30) of cases. Vascular invasion was identified in 8.0% (2/25), perineural invasion in 45.0% (9/20), and cartilage invasion in 54.2% (13/24) of cases. High-grade transformation was observed in 30.0% (3/30) of cases. The median Ki67 proliferation index was 10%, with an interquartile range of 5–30%, and 95.83% (23/24) of patients were P63-positive. The relationship between clinicopathological features and prognosis is summarized in Table 2.

**Table 2:** Correlation between clinicopathologic characteristics and prognosis of 30 LHACC patients(n) with Fisher’s exact test *P*-value

Factor	State	Lymph node metastasis cN (clinical N)			Nerves invasion			Vascular invasion			Cartilaginous invasion			High-grade transformation			Pathological grading				Ki67%			P63		
		N0	N1-2	P	No	Yes	P	No	Yes	P	No	Yes	P	No	Yes	P	1	2	3	P	< 15%	≥ 15%	P	-	+	P
Survival	Alive	22	2	0.501	10	6	0.217	19	2	0.700	10	10	0.363	22	2	0.501	13	5	4	/	15	9	0.455	1	18	0.792
	Deceased	5	1		1	3		4	0		1	3		5	1		1	0	1		3	3		0	5	
Locoregional recurrence	NO	22	3	0.567	9	8	0.579	19	2	0.700	10	10	0.363	24	1	0.064	13	4	2	/	15	10	0.696	1	20	0.875
	YES	5	0		2	1		4	0		1	3		3	2		1	1	3		3	2		0	3	
Distant metastasis	NO	15	1	0.586	9	3	0.040*	12	2	0.303	7	7	0.473	15	1	0.448	9	1	3	/	11	5	0.251	1	13	0.583
	YES	12	2		2	6		11	0		4	6		12	2		5	4	2		7	7		0	10	

^*^P < 0.05, **P < 0.01. Values in bold indicate statistically significant differences (P < 0.05).

Preoperative imaging (contrast-enhanced CT and/or MRI) identified suspicious cervical lymph nodes in 3 patients (10.0%). Overall, 11 patients underwent neck dissection, including 3 therapeutic procedures performed for clinically or radiologically positive nodes and 8 elective procedures undertaken at the surgeon’s discretion during total laryngectomy in the presence of paralaryngeal tissue involvement or enlarged lymph nodes. Pathologic analysis confirmed nodal metastasis in only one patient, who exhibited multi-level involvement (left level IIB: 3/4; right level IIB: 4/8; right level IV: 1/8; right level V: 1/3). No statistically significant association was observed between lymph node metastasis and survival status (Fisher’s exact test, *P* > 0.05). Detailed nodal findings are provided in Online Resource 8.

#### Neural invasion patterns

Detailed neural invasion data were available for 20 patients. Of the 9 patients with detailed records of neural invasion, 2 (22.2%) showed involvement of the recurrent laryngeal nerve and 1 (11.1%) of the external branch of the superior laryngeal nerve. These patients presented with either hoarseness or pharyngeal foreign-body sensation as the initial symptom; however, no definite correlation between specific nerve invasion and presenting symptoms could be established, likely due to the limited sample size and the complex neural anatomy of the laryngopharynx.

Due to the complex microanatomy of laryngeal nerves, the remaining cases were classified as generalized neural invasion. Symptoms of hoarseness were observed in 66.7% (6/9) of patients, often accompanied by reduced mobility of one or both vocal cords. Details on patient age, sex, staging, tumor location, nerve involvement, clinical symptoms, vocal cord mobility, prognosis, and tumor size are summarized in Table 3. Perineural invasion was significantly associated with distant metastasis (Fisher’s exact test, *P* = 0.040, OR = 9.00, 95% CI: 1.14–71.01), supporting its role as a marker of aggressive tumor behavior. Observed patterns suggested that tumor location may influence pathological aggressiveness and prognosis.

**Table 3:** Specific features of nerve invasion in the patient’s throat and pharynx

Patient/age/gender	Tumor size	Staging	Location	Invaded nerve	Symptoms	Vocal cord mobility	Prognosis
1/23/F	6.0*5.0*1.5 cm	Stage Ⅲ	Subglottic	Recurrent laryngeal nerve	Hoarseness, foreign body sensation in the throat	Bilateral vocal cords mobile	Pulmonary metastasis
2/35/F	0.8*0.7*0.3 cm	Stage Ⅱ	Subglottic	Nerve	Reflexive coughing and sputum production	Slightly reduced mucosal wave, bilateral vocal cords mobile	Tongue base recurrence
3/50/F	1.3*0.8*0.5 cm	Stage Ⅲ	Subglottic	Nerve	Hoarseness	Slightly restricted mobility in both vocal cords	Pulmonary metastasis
4/34/F	2.5*1.6*1.3 cm	Stage Ⅳ	Supraglottic	Nerve	Hoarseness, throat pain, stridor during movement, choking while drinking	Left vocal cord restricted, right vocal cord normal	Pulmonary metastasis, death
5/76/F	3.0*2.5*1.5cm	Stage Ⅲ	Subglottic	Right recurrent laryngeal nerve, multifocal nerve invasion observed in tracheal tumor	Throat foreign body sensation, hoarseness, shortness of breath after activity	Bilateral vocal cords mobile	No locoregional recurrence or metastasis
6/58/M	1.5*0.6*0.4 cm	Stage Ⅳ	Supraglottic	Nerve	Throat foreign body sensation, swallowing pain, worsening hoarseness	Mucosal wave absent, left hemilarynx immobile, right hemilarynx restricted	No locoregional recurrence or metastasis, death
7/55/F	3.2*3*1.2 cm	Stage Ⅲ	Postcricoid region	Perineural and neural invasion	Unprovoked throat pain, radiating to ear, choking while swallowing	Normal bilateral vocal cord mobility	No locoregional recurrence
8/52/M	2.7*2.1*2cm	Stage Ⅳ	Postcricoid region	Nerve	Throat foreign body sensation	Left vocal cord restricted, right vocal cord normal	Pulmonary metastasis
9/49/M	NA	Stage Ⅳ	Postcricoid region	Left superior laryngeal nerve	Hoarseness, breathing difficulty	Bilateral vocal cords fixed near midline	Pulmonary metastasis

### Survival and prognostic risk factors

The median follow-up was 36 months (range, 4–130 months), with 9 censored patients who had follow-up shorter than 3 or 5 years without tumor progression or death. The 3-year, 5-year, and 10-year OS rates for the patients were 95.5, 76.4, and 38.2%, respectively. DFS rates were 79.2, 49.4, and 10.3%. Due to the limited follow-up period, 16 patients were followed up to 5 years (including those who died within this period). Among these, the 3-year and 5-year OS rates were 93.8 and 75.0%, and the DFS rates were 75.0 and 50.0%, respectively.

**Fig. 2:**
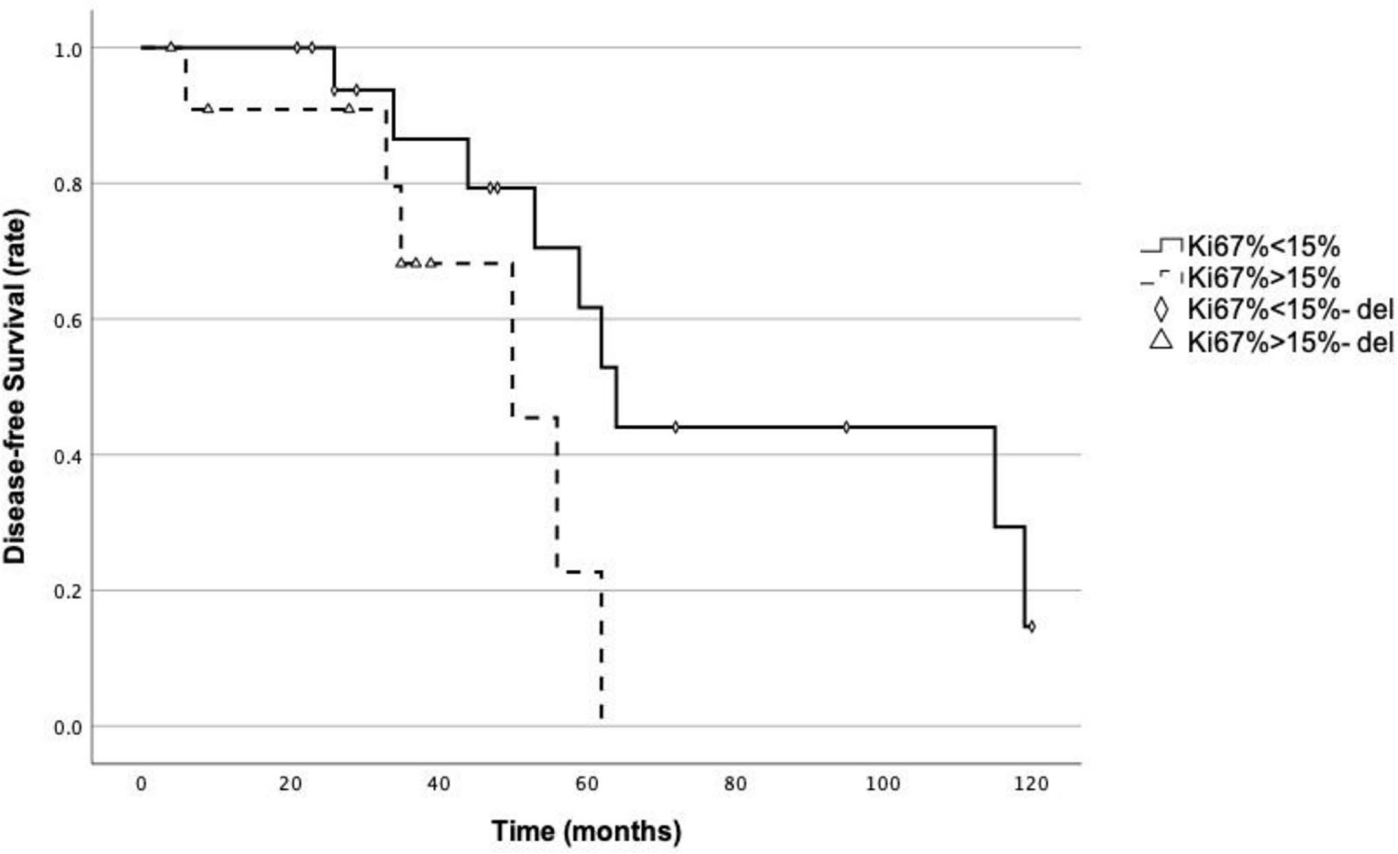
Ki-67 proliferation index (%) and disease-free survival stratification curve (*P* = 0.041)

Univariate survival analysis indicated that none of the 15 factors included in the analysis showed statistically significant correlation with overall survival (*P* > 0.05, see Online Resource 9). Among the evaluated factors, only a Ki-67 proliferation index greater than 15% was significantly associated with poorer DFS (*P* = 0.041; Fig. 2). For patients with Ki-67 proliferation index < 15%, the mean survival time was 80.2 months, with a median survival time of 64 months (95% CI: 55.984–72.016). For those with Ki-67 proliferation index ≥ 15%, the mean survival time was 46.455 months, with a median survival time of 50 months (95% CI: 30.808–69.192).

Postoperative radiotherapy (PORT) improved the 5-year overall survival (OS) rate (81.8% vs. 65.6%), although the difference was not statistically significant (*P* = 0.661). Five patients (16.7%) developed locoregional recurrence, all of whom had received PORT (*P* = 0.098, Fisher’s exact test). These patients predominantly exhibited high-risk pathological features—such as advanced T classification or positive margins—that likely predisposed them to recurrence despite adjuvant treatment. Because all recurrent cases ultimately progressed, Kaplan–Meier analysis of local recurrence-free survival was not feasible. All recurrent cases occurred in patients who received PORT and exhibited high-risk pathological features. Larger cohorts are needed to confirm these results.

### Genomic alterations

**Fig. 3:**
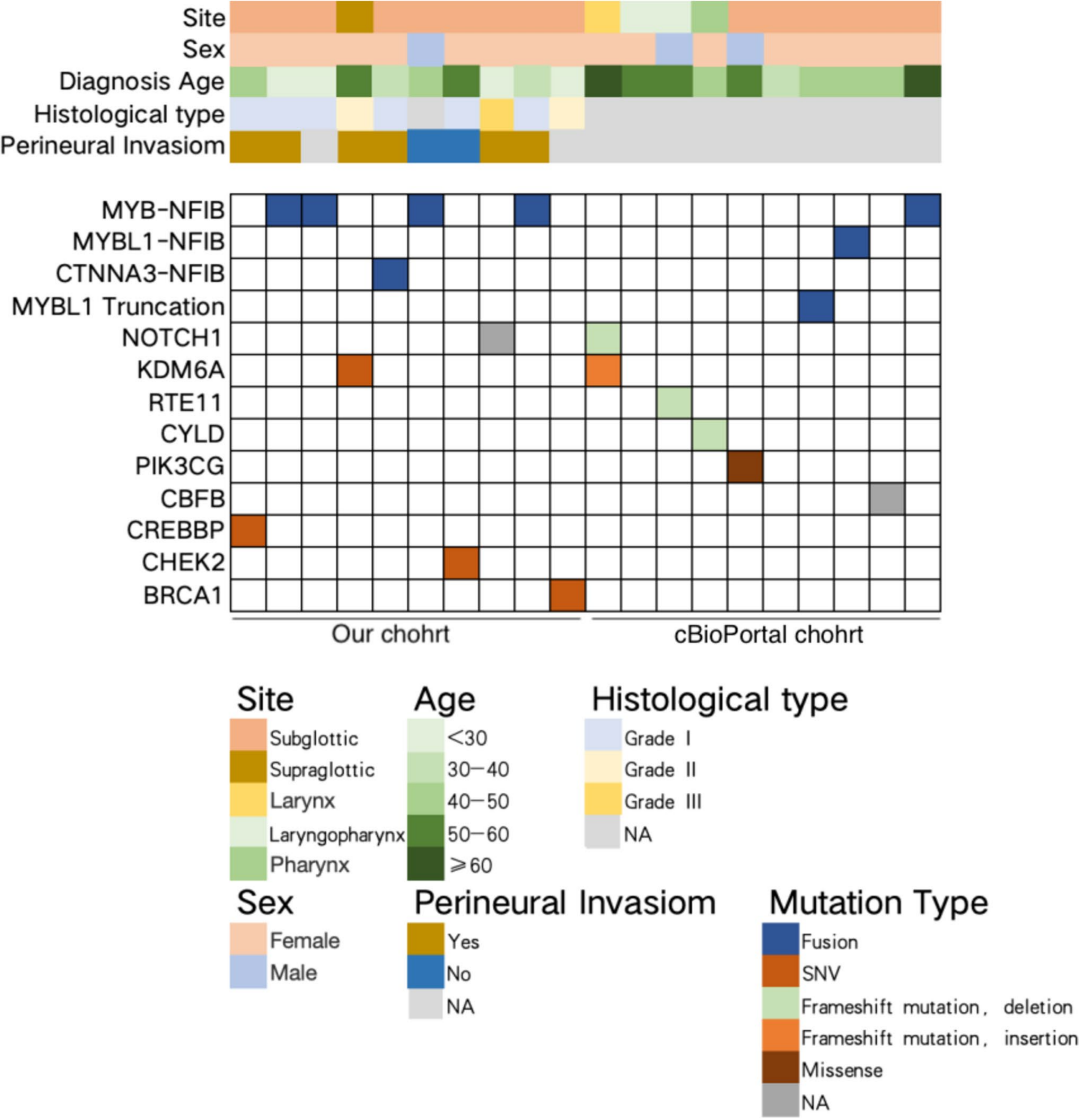
Patient information and genetic mutation data from this study and cBioPortal database

Genetic testing of our cohort revealed that 40% (4/10) of patients harbored MYB-NFIB fusion (Online Resource 10). These data were further combined with 10 laryngeal adenoid cystic carcinoma cases from the cBioPortal for Cancer Genomics database to generate a waterfall plot summarizing genetic alterations, histological grading, perineural invasion status, and patient demographics (Fig. 3).

Analysis of the combined dataset showed a predominance of female patients, with ages concentrated between 30 and 50 years. Histological grades ranged from I to III, and perineural invasion was relatively frequent. Common genetic alterations included MYB-NFIB and MYBL1-NFIB fusions, as well as NOTCH1, PIK3CG, and KDM6A mutations. Perineural invasion and high-grade tumors were more frequent in patients harboring MYB-NFIB fusion, PIK3CG, or NOTCH1 mutations, suggesting a potential association between these genetic alterations and aggressive tumor behavior.

These genomic findings provide a molecular context that may help explain the observed patterns of perineural invasion and aggressive clinical behavior and will be further explored in relation to patient outcomes in the following sections.

## Discussion

### Epidemiology and clinical differences

Squamous cell carcinoma (SCC) is the most common malignant tumor of the larynx, accounting for more than 90% of cases. It predominantly arises in the glottic and supraglottic regions, whereas subglottic SCC is rare. SCC occurs more frequently in men, with smoking showing a clear dose–response relationship.

In contrast, adenoid cystic carcinoma (ACC) of the laryngopharynx is exceedingly rare, representing less than 1% of malignant laryngeal tumors (Dubal et al. 2015). Unlike SCC, which often manifests as exophytic masses or ulcerations, ACC typically presents as a slowly growing submucosal mass characterized by infiltration into adjacent tissues, perineural spread, and occasional misdiagnosis as hypertrophic laryngitis due to overlapping clinical symptoms (Moukarbel et al. 2008).

The metastatic patterns of these two malignancies are also distinct. Lymph node metastasis is a common early feature of laryngeal SCC, particularly in supraglottic tumors owing to their rich lymphatic drainage (Johnson et al. 2020; Lian et al. 2013). By contrast, glottic SCC has a lower nodal spread risk, while supraglottic SCC demonstrates earlier recurrence and poorer outcomes (Sanabria et al. 2020; Gupta et al. 2021; Haapaniemi et al*.* 2017). Most recurrences occur within three years of treatment, underscoring the need for intensive short-term surveillance. Conversely, LHACC is characterized by rare nodal involvement (most often in levels IB and II) but a strong propensity for hematogenous metastasis—most frequently to the lungs, bones, and liver (Min et al. 2012). Its indolent yet relentless progression necessitates long-term follow-up well beyond the surveillance window typical for SCC.

These biological differences translate into distinct prognostic patterns. The incidence of distant metastasis in SCC is relatively low (10–20%), but rises in advanced or recurrent disease, most often affecting the lungs, followed by bones, liver, brain, and adrenal glands. In contrast, distant metastasis occurs in 20–55% of LHACC patients within 10 years, with the lungs accounting for ~ 80% of cases. Survival data further highlight the disparity: while poorly differentiated SCC carries a 5-year overall survival of approximately 50%, high-grade transformation (HGT) fares worse, with 5-year survival around 38.7% (Shi et al. 2014; Mur et al. 2022; Zhu et al. 2022). Importantly, 5-year survival underestimates the long-term burden of LHACC, since late recurrences and metastases are frequent.

Compared with adenoid cystic carcinomas (ACCs) of the salivary glands or sinonasal tract, laryngopharyngeal ACC (LHACC) displays distinct epidemiologic, clinicopathologic, and prognostic characteristics. More than 90% of head and neck ACCs arise from the major or minor salivary glands, whereas sinonasal and laryngopharyngeal cases are relatively rare. (Michel et al. 2013) Clinically, site-specific symptoms reflect anatomic context: sinonasal tumors often present with nasal obstruction, facial numbness, or visual disturbances, while LHACC typically manifests as hoarseness or pharyngeal discomfort due to superior laryngeal nerve involvement. (Amit et al. 2013; De Morais et al. 2023) Pathologically, sinonasal ACCs frequently show extensive perineural invasion (> 50%) and skull base extension, particularly in ethmoidal and sphenoidal lesions, whose 5-year disease-free survival drops to approximately 25% compared with about 83% for maxillary sinus tumors. (Amit et al. 2013) Prognostically, salivary gland ACCs generally have the most favorable outcomes, sinonasal ACCs the worst, and LHACC shows intermediate survival, likely reflecting its relatively accessible surgical anatomy. (Lukšić et al. 2016).

Consistent with these observations, our cohort demonstrated that LHACC exhibited slow but invasive growth, strong perineural tropism, and a high incidence of late distant metastasis. These findings align with previous reports while providing new insights from the largest single-institution dataset to date.

### Clinicopathological characteristics

Among the variables analyzed, proliferation index and invasion patterns emerged as the most informative markers differentiating LHACC from laryngeal and hypopharyngeal squamous cell carcinoma (LHSCC).

#### Proliferation marker (Ki-67)

The Ki-67 index was identified as a significant predictor of disease-free survival (DFS). In our cohort, higher Ki-67 levels (> 15%) were associated with significantly worse outcomes, consistent with prior studies showing that elevated Ki-67 expression correlates with increased tumor aggressiveness (Nordgård et al. 1997).

#### Lymph node metastasis

With only a single nodal metastasis event, the study is intrinsically limited by insufficient statistical power, constraining both categorical testing and time-to-event modeling. Under such conditions, the non-significant findings primarily reflect methodological limitations rather than a definitive lack of prognostic effect. Accordingly, the prognostic role of cervical lymph node involvement should remain an open question, warranting validation in studies with larger sample sizes or pooled institutional cohorts.

#### Neural invasion

Neural invasion is rare in SCC, typically limited to small peritumoral nerves and seldom involving the recurrent or superior laryngeal nerves—even in advanced disease (Jaiswal et al*.* 2023; Zhu et al. 2021). By contrast, it is a hallmark feature of LHACC, with previous reports showing incidences as high as 90% (Ganly et al. 2006). In our study, neural invasion was observed in 39.1% (9/23) of cases at initial surgery. The subglottic location of LHACC facilitates extralaryngeal spread through the cricothyroid membrane and adjacent tissues, leading to extensive perineural and cartilage invasion (Hogg et al. 1999). Notably, although neural invasion was associated with a higher mortality proportion on χ^2^ analysis, this difference was not significant on Kaplan–Meier curves. This likely reflects limited sample size and event numbers, where χ^2^ may detect endpoint differences that the time-dependent log-rank test cannot. Thus, the prognostic impact of neural invasion on survival requires confirmation in larger cohorts.

In our cohort, two cases involved the recurrent laryngeal nerve (RLN), and one involved the superior laryngeal nerve (SLN). Functionally, hoarseness is more likely related to RLN involvement, whereas pharyngeal foreign-body sensation may reflect invasion of the internal branch of the SLN. However, no definite correlation between specific nerve invasion and presenting symptoms could be established, likely due to the limited sample size and the complex neural anatomy of the laryngopharynx. Compared with other head and neck subsites, ACC arising in regions closer to the skull base (such as the nasopharynx, nasal cavity, and maxilla) is associated with a higher risk of local recurrence. This is largely explained by the propensity for perineural spread toward the intracranial space and the surgical limitations in obtaining negative margins adjacent to critical neurovascular structures (Samuel et al*.* 2017). In contrast, while LHACC also demonstrates a high incidence of perineural invasion, the anatomic accessibility of the larynx generally allows for a higher likelihood of achieving adequate surgical margins, which may partially account for the relatively lower local recurrence rates observed in this site.

#### Clinical correlates

These anatomic and pathological features translate into distinctive clinical symptoms. In our cohort, hoarseness and throat discomfort or pain were common presenting symptoms. Notably, hoarseness was significantly associated with poorer overall survival. Further analysis revealed that patients presenting with hoarseness had significantly larger tumors (median 1.8 cm [IQR 0.6–2.3] vs 1.1 cm [IQR 0.7–1.8], *P* = 0.04), tended to have more advanced T stages (T4: 42.9% vs 25.0%, *P* = 0.33), and exhibited a higher frequency of perineural invasion (60.0% vs 30.0%, *P* = 0.38). Although the latter two differences did not reach statistical significance, these consistent trends suggest that hoarseness may serve as a clinical indicator of greater tumor burden and potential neural involvement. This finding aligns with the biological plausibility that laryngeal adenoid cystic carcinoma involving the recurrent or superior laryngeal nerves often presents with voice changes, reflecting tumor extension beyond the submucosa into adjacent neurovascular structures. This observation is consistent with the previous reports by Pukander et al. (Pukander 2000) and Zhang et al. (Zhang, 2019), who described frequent invasion of the recurrent or superior laryngeal nerves in up to 50% of LHACC cases, with corresponding variability in symptoms depending on the affected branch.

### Surgical strategy, margin status, and postoperative radiotherapy in laryngeal and hypopharyngeal adenoid cystic carcinoma

While tumor biology is critical, surgical decision-making and margin status ultimately shape long-term outcomes. In LHACC, the choice of surgical procedure is guided by tumor location, local invasion, and the need to preserve laryngeal function. Given the submucosal spread and propensity for perineural invasion, total laryngectomy is generally recommended to achieve complete R0 resection. By contrast, in laryngeal squamous cell carcinoma (SCC), the choice of surgical procedure largely depends on tumor size. Smaller tumors are often amenable to partial laryngectomy or organ-preserving approaches, whereas larger tumors typically require total laryngectomy to achieve adequate local control, in accordance with current clinical practice guidelines (Pfister et al. 2020).

In our cohort, cervical lymph node metastasis was uncommon, occurring in only one patient despite 11 neck dissections. This low rate aligns with the reported 6–10% incidence in head and neck ACC (Amit et al. 2015). Considering the typical pattern of perineural and distant hematogenous spread, the role of elective neck dissection in LHACC appears limited. Our findings support the prevailing recommendation that neck dissection should be performed primarily for clinically or radiologically suspicious lymph nodes rather than as a routine prophylactic procedure.

Regarding adjuvant treatment, 19 of 30 patients (63.3%) received postoperative radiotherapy (PORT). The 5-year overall survival was higher in the PORT group than in the surgery-alone group (81.8% vs. 65.6%), although this difference was not statistically significant (*P* = 0.661). All five patients who developed local recurrence had received PORT. These patients were characterized by high-risk disease, including advanced primary tumors (T3–T4 stage) and adverse pathological features such as positive margins or extensive local invasion. Furthermore, four of the five recurrences occurred in the paratracheal or thyroid-adjacent regions, where delivering adequate radiation doses is often challenging due to the proximity of critical structures and the potential for functional impairment. Therefore, these recurrences likely reflect anatomical dose limitations rather than intrinsic radio-resistance, rather than indicating inherent treatment failure.

Nevertheless, given these anatomical challenges and the risk of local recurrence, local control in LHACC is dose-dependent. Postoperative radiotherapy (PORT) doses below 60 Gy have been associated with higher recurrence risk (Chen et al. 2006; Ogino et al.^1995^). Achieving R0 resection combined with adequately dosed PORT remains central to improving outcomes (Chen et al. 2024; Rafi et al. 2023). Proton beam therapy (PBT) may help overcome anatomical dose constraints, as it can deliver sufficient dose to the target in anatomically constrained regions while minimizing exposure to surrounding healthy tissues (Nakamura et al. 2024).

Importantly, all recurrences in our cohort occurred in PORT-treated patients. This observation likely reflects treatment-selection bias, as PORT was primarily administered to high-risk cases. Given the small sample size, larger, risk-balanced studies are needed to more accurately assess the effect of PORT on local control in LHACC.

### Molecular alterations and prognostic implication

Beyond pathological factors, the indolent yet persistent progression of LHACC may be attributed to its distinct molecular background. LHACC and laryngeal SCC exhibit markedly different molecular profiles. SCC is primarily characterized by TP53 mutations, often occurring early in tumorigenesis and associated with tumor aggressiveness, along with EGFR overexpression, which contributes to tumor growth, invasiveness, and treatment response (Kim et al. 2023).

In contrast, the hallmark molecular feature of LHACC is the MYB-NFIB fusion gene, detected in over 70% of cases, particularly in salivary gland-derived ACCs (Ferrarotto et al. 2016). ACCs originating from smaller glands, such as the hard palate, soft palate, and trachea, exhibit more diverse fusion forms, including atypical fusions like MYB-TGFBR3 and MYBL1-NFIB (Mitani et al. 2016). In our cohort, MYB-NFIB was the predominant fusion. MYB regulates genes involved in apoptosis, cell cycle control, adhesion, growth, differentiation, and angiogenesis, highlighting its central role in tumor biology. Additionally, Notch-1 positivity increases with tumor stage, correlating with adjacent tissue invasion in ACC (Xu et al. 2019). These molecular insights underscore that both biological and surgical determinants must be considered when interpreting the clinical course of LHACC. The clinical course of LHACC is influenced by its molecular characteristics. In our cohort, MYB-NFIB fusion was the most prevalent alteration. Huang et al. reported that high MYB TSS2 activity is associated with metastasis, drug resistance, and aggressive tumor behavior (Huang et al. 2024), while Zisis et al. highlighted that molecular marker, including MYB-NFIB and NOTCH pathway alterations, can complement histological subtypes in prognosis assessment (Zisis et al. 2025). Due to limited patient numbers and incomplete long-term data, we provide a descriptive overview of these molecular findings, emphasizing their potential relevance for tumor behavior and prognosis. Moreover, genetic data were available for only 10 LHACC cases, and therefore conclusions drawn from the cBioPortal for Cancer Genomics database remain limited.

This study has limitations, including one loss to follow-up among 30 patients and potential recall bias from the long follow-up period, which may affect the accuracy of treatment-related data. Variations in pathological reporting over time led to some missing data. Future studies should include secondary pathological review to clarify subtype proportions and clinical significance.

## Conclusion

In conclusion, hoarseness at presentation, perineural invasion, and a high Ki-67 proliferation index (> 15%) emerged as significant prognostic indicators in LHACC. Hoarseness likely reflects greater tumor burden and neural involvement, while perineural invasion denotes aggressive biological behavior predisposing to distant spread. Elevated Ki-67 expression was associated with shorter disease-free survival, highlighting the impact of proliferative activity on outcome. These findings underscore the importance of early recognition, meticulous pathological assessment, and individualized adjuvant therapy for high-risk patients. Given the tumor’s indolent yet persistent course, long-term surveillance remains essential. Further multicenter and molecular studies are warranted to validate these markers and optimize management strategies.

## Supplementary Information

Below is the link to the electronic supplementary material.

Supplementary file1. Locoregional recurrence and distant metastasis of patients with adenoid cystic carcinoma of laryngeal

Supplementary file2. Specific findings on laryngoscopy in 21 patients

Supplementary file3. A soft tissue mass shadow was observed on the right vocal cord and beneath it, showing iso-T1 signal

Supplementary file4. A soft tissue mass shadow was observed on the right vocal cord and beneath it, showing prolonged T2 signal

Supplementary file5. With a narrowed laryngeal cavity. The soft tissue at the base of the tongue appeared thickened, exhibiting iso-T1 signal

Supplementary file6. With a narrowed laryngeal cavity. The soft tissue at the base of the tongue appeared thickened, exhibiting iso-T2 signal

Supplementary file7. With a narrowed laryngeal cavity. The soft tissue at the base of the tongue appeared thickened, with marked enhancement after contrast administration

Supplementary file8. Lymph node dissection site and lymph node metastasis in 11 patients with laryngeal adenoid cystic carcinoma

Supplementary file9. Univariate Log-rank Survival Analysis of 30 Patients

Supplementary file10. Subgroup mutated genes, nucleotide mutations, amino acid mutations, mutation types, mutation abundance, TMB information

## Data Availability

The datasets generated and/or analyzed during the current study are available from the corresponding author on reasonable request.
