# Supplementary material for: Clinicopathological features and prognostic analysis of 30 patients with laryngeal and hypopharyngeal adenoid cystic carcinoma: a single-center retrospective study
Source: J Cancer Res Clin Oncol. 2026 Apr 8;152(4):84. doi: 10.1007/s00432-026-06449-1 (PMC13062074; doi:10.1007/s00432-026-06449-1)
Supplement: Supplementary file 1 — Supplementary file1. Locoregional recurrence and distant metastasis of patients with adenoid cystic carcinoma of laryngeal [file 432_2026_6449_MOESM1_ESM.zip › Online Resource 1.docx]

| **Observation index** | **n (%)** |
| --- | --- |
| Recurrence site |  |
| Trachea & Thyroid gland  Thyroid gland  Tracheostoma  Nasopharynx  Tracheostoma & Thyroid gland | 1(0.03)  1(0.03)  1(0.03)  1(0.03)  1(0.03) |
| No recurrence | 24（0.80） |
| Metastasis site  Lung  Lung & Liver  No metastasis | 15(0.50)  1(0.03)  14(0.47) |

**Corresponding Author**:
**Xiaohong Chen, M.D.**
Department of Otolaryngology Head and Neck Surgery,
Beijing Tongren Hospital, Capital Medical University
Key Laboratory of Otolaryngology Head and Neck Surgery (Capital Medical University), Ministry of Education
1 Dongjiaominxiang Street, Dongcheng District,
Beijing 100730, P.R. China
Mobile: +86 13911071002
Email: [trchxh@163.com](mailto:trchxh@163.com)
ORCID: [https://orcid.org/0000-0002-3825-2647](https://orcid.org/0000-0002-3825-2647)
