# Supplementary material for: Clinicopathological features and prognostic analysis of 30 patients with laryngeal and hypopharyngeal adenoid cystic carcinoma: a single-center retrospective study
Source: J Cancer Res Clin Oncol. 2026 Apr 8;152(4):84. doi: 10.1007/s00432-026-06449-1 (PMC13062074; doi:10.1007/s00432-026-06449-1)
Supplement: Supplementary file 2 — Supplementary file2. Specific findings on laryngoscopy in 21 patients [file 432_2026_6449_MOESM2_ESM.zip › Online Resource 2.docx]

| **Location** | **Tumor Morphology** | **Additional Description** |
| --- | --- | --- |
| **Subglottic** | Broad-based protrusion |  |
| **Subglottic, Posterior Wall of Upper Trachea** | Pale red bulge |  |
| **Glottic area, Subglottic, Bilateral Vocal Cord Margins** | Broad-based, rough mass |  |
| **Supraglottic** | Protruding irregular mass |  |
| **Glottic margin and Subglottic** | Broad-based protrusion |  |
| **Supraglottic** | Broad-based protrusion | Surface irregular |
| **Vocal Cord Lower Margin** | Broad-based protrusion | Pale red, with noticeable vascular dilation extending to the subglottic |
| **Lower Margin of Vocal Cord** | Broad-based protrusion |  |
| **Broad-based protrusion** | Broad-based protrusion |  |
| **Surface of Vocal Cord** | Hematoma | Edematous mucosa at edges |
| **Subglottic** | Pinkish-red mass |  |
| **Posterior Glottic Area** | Protruding mass |  |
| **Right Vocal Cord and Subglottic Margin** | Broad-based protrusion |  |
| **Supraglottic** | Broad-based protrusion |  |
| **Posterior Left Subglottic Area** | Broad-based protrusion |  |
| **Posterior Left Subglottic Area** | Broad-based protrusion |  |
| **Subglottic** | Locally irregular protrusion | Subglottic narrowing |
| **Bilateral Vocal Cords, Subglottic** | Broad-based, rough mass |  |
| **Posterior Subglottic Area** | Broad-based, rough mass |  |
| **Left Vocal Cord and Subglottic** | Broad-based protrusion |  |
| **Post-cricoid Area** | Broad-based, rough mass | Resembling granulation tissue and pseudo- membrane |
| **Post-cricoid Area and the left arytenoid region** | Broad-based protrusion | Involvement of the left piriform sinus |

**Corresponding Author**:
**Xiaohong Chen, M.D.**
Department of Otolaryngology Head and Neck Surgery,
Beijing Tongren Hospital, Capital Medical University
Key Laboratory of Otolaryngology Head and Neck Surgery (Capital Medical University), Ministry of Education
1 Dongjiaominxiang Street, Dongcheng District,
Beijing 100730, P.R. China
Mobile: +86 13911071002
Email: [trchxh@163.com](mailto:trchxh@163.com)
ORCID: [https://orcid.org/0000-0002-3825-2647](https://orcid.org/0000-0002-3825-2647)
