# Supplementary figures and images for: Clinicopathological features and prognostic analysis of 30 patients with laryngeal and hypopharyngeal adenoid cystic carcinoma: a single-center retrospective study
Source: J Cancer Res Clin Oncol. 2026 Apr 8;152(4):84. doi: 10.1007/s00432-026-06449-1 (PMC13062074; doi:10.1007/s00432-026-06449-1)

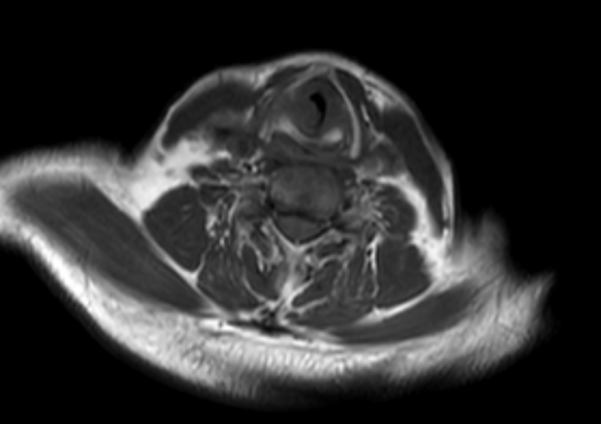

Supplement: Supplementary file 3 — Supplementary file3. A soft tissue mass shadow was observed on the right vocal cord and beneath it, showing iso-T1 signal [file 432_2026_6449_MOESM3_ESM.png]

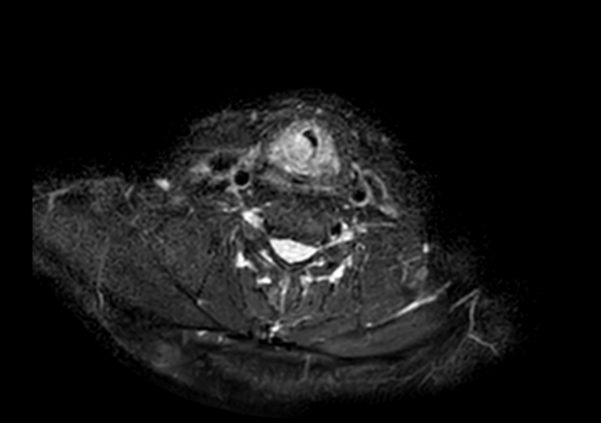

Supplement: Supplementary file 4 — Supplementary file4. A soft tissue mass shadow was observed on the right vocal cord and beneath it, showing prolonged T2 signal [file 432_2026_6449_MOESM4_ESM.png]

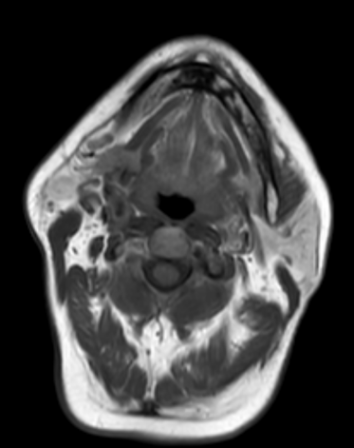

Supplement: Supplementary file 5 — Supplementary file5. With a narrowed laryngeal cavity. The soft tissue at the base of the tongue appeared thickened, exhibiting iso-T1 signal [file 432_2026_6449_MOESM5_ESM.png]

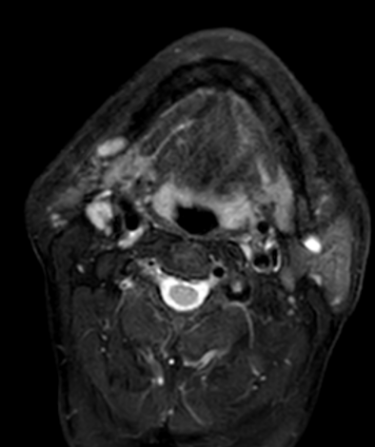

Supplement: Supplementary file 6 — Supplementary file6. With a narrowed laryngeal cavity. The soft tissue at the base of the tongue appeared thickened, exhibiting iso-T2 signal [file 432_2026_6449_MOESM6_ESM.png]

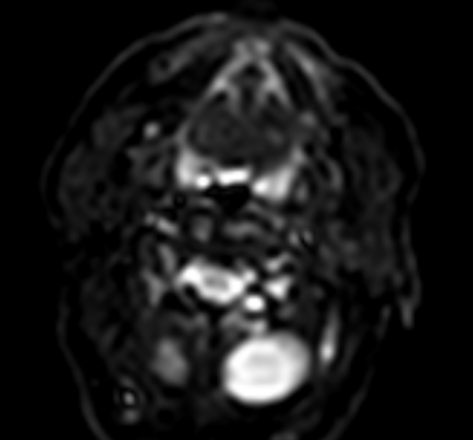

Supplement: Supplementary file 7 — Supplementary file7. With a narrowed laryngeal cavity. The soft tissue at the base of the tongue appeared thickened, with marked enhancement after contrast administration [file 432_2026_6449_MOESM7_ESM.png]
