# Supplementary material for: Clinicopathological features and prognostic analysis of 30 patients with laryngeal and hypopharyngeal adenoid cystic carcinoma: a single-center retrospective study
Source: J Cancer Res Clin Oncol. 2026 Apr 8;152(4):84. doi: 10.1007/s00432-026-06449-1 (PMC13062074; doi:10.1007/s00432-026-06449-1)
Supplement: Supplementary file 8 — Supplementary file8. Lymph node dissection site and lymph node metastasis in 11 patients with laryngeal adenoid cystic carcinoma [file 432_2026_6449_MOESM8_ESM.zip › Online Resource 8.docx]

| Initial Neck Lymph Node Dissection | Ⅰ-Ⅵ | Ⅲ、Ⅳ | Ⅱ、Ⅲ | pre-laryngeal lymph nodes | Ⅱ-Ⅴ | tracheoesophageal groove and right II | Ⅱ、Ⅲ、Ⅵ | Ⅱ、Ⅲ、Ⅳ | Ⅱ | pre-tracheal lymph nodes, Ⅳ | pre-laryngeal lymph nodes |
| --- | --- | --- | --- | --- | --- | --- | --- | --- | --- | --- | --- |
| Lymph node metastasis  pN（pathologic N） | No | No | No | No | Yes | No | No | No | No | No | No |

**Corresponding Author**:
**Xiaohong Chen, M.D.**
Department of Otolaryngology Head and Neck Surgery,
Beijing Tongren Hospital, Capital Medical University
Key Laboratory of Otolaryngology Head and Neck Surgery (Capital Medical University), Ministry of Education
1 Dongjiaominxiang Street, Dongcheng District,
Beijing 100730, P.R. China
Mobile: +86 13911071002
Email: [trchxh@163.com](mailto:trchxh@163.com)
ORCID: [https://orcid.org/0000-0002-3825-2647](https://orcid.org/0000-0002-3825-2647)
