# Supplementary material for: Clinicopathological features and prognostic analysis of 30 patients with laryngeal and hypopharyngeal adenoid cystic carcinoma: a single-center retrospective study
Source: J Cancer Res Clin Oncol. 2026 Apr 8;152(4):84. doi: 10.1007/s00432-026-06449-1 (PMC13062074; doi:10.1007/s00432-026-06449-1)
Supplement: Supplementary file 9 — Supplementary file9. Univariate Log-rank Survival Analysis of 30 Patients [file 432_2026_6449_MOESM9_ESM.zip › Online Resource 9.docx]

| Factor | n | Survival rate (100%) | | | χ^2^ | P |
| --- | --- | --- | --- | --- | --- | --- |
|  |  | 1 year | 3 years | 5 years |  |  |
| Gender | | | | | | |
| Male  Female | 10  20 | 1.000  1.000 | 0.833±0.152  1.000 | 0.833±0.152  0.750±0.125 | 0.78 | 0.378 |
| Age(years) | | | | | | |
| ≥ 60  ＜60 | 4  26 | 1.000  1.000 | 0.401±0.207  0.500±0.354 | 0.401±0.207  0.500±0.354 | 0.91 | 0.339 |
| Primary site | | | | | | |
| Supraglottic  Subglottic  Post-cricoid area | 4  23  3 | 1.000  1.000  1.000 | 0.667±0.272  1.000  1.000 | 0.333±0.272  0.917±0.080  0.000 | 5.1 | 0.079 |
| Clinical stage | | | | | | |
| Ⅱ  Ⅲ  Ⅳ | 11  9  10 | 0.833±0.152  0.800±0.179  0.000 | 0.833±0.152  0.800±0.179  0.000 | 0.833±0.152  0.800±0.179  0.000 | 2.89 | 0.236 |
| Pathological grading | | | | | | |
| Level 1  Level 2  Level 3 | 14  5  5 | 0.800±0.179  1.000  0.000 | 0.800±0.179  1.000  0.000 | 0.800±0.179  1.000  0.000 | 0.41 | 0.814 |
| Lymph node metastasis | | | | | | |
| N0  N1-2 | 27  3 | 1.000  0.667±0.272 | 0.393±0.204  0.667±0.272 | 0.393±0.204  0.667±0.272 | 1.33 | 0.250 |
| Nerves invasion | | | | | | |
| Yes  No | 9  11 | 0.000  0.500±0.354 | 0.000  0.500±0.354 | 0.000  0.500±0.354 | 1.54 | 0.214 |
| Vascular invasion | | | | | | |
| Yes  No | 2  23 | 1.000  0.933±0.064 | 1.000  0.420±0.217 | 1.000  0.420±0.217 | 0.13 | 0.715 |
| Cartilaginous invasion | | | | | | |
| Yes  No | 13  11 | 0.500±0.230  0.000±0.000 | 0.500±0.230  0.000±0.000 | 0.500±0.230  0.000±0.000 | 0.15 | 0.703 |
| High-grade transformation | | | | | | |
| Yes  No | 3  27 | 0.000  1.000 | 0.000  0.499±0.217 | 0.000  0.499±0.217 | 0.04 | 0.847 |
| Pathological grading | | | | | | |
| Level 1  Level 2  Level 3 | 14  5  5 | 0.800±0.179  1.000  0.000 | 0.800±0.179  1.000  0.000 | 0.800±0.179  1.000  0.000 | 0.41 | 0.814 |
| Ki67% | | | | | | |
| ＜15%  ≥15% | 18  12 | 0.836±0.108  0.000 | 0.557±0.239  0.000 | 0.557±0.239  0.000 | 0.991 | 0.320 |
| P63 | | | | | | |
| +  - | 23  1 | 0.467±0.208  1.000 | 0.467±0.208  1.000 | 0.467±0.208  1.000 | 0.07 | 0.796 |
| Margin | | | | | | |
| Positive  Negative | 8  15 | 0.000  0.889±0.105 | 0.000  0.889±0.105 | 0.000  0.889±0.105 | 1.84 | 0.175 |
| Initial Treatment | | | | | | |
| Surgery  Surgery & Adjuvant therapy | 10  20 | 0.572±0.249  0.833±0.108 | 0.572±0.249  0.278±0.230 | 0.572±0.249  0.278±0.230 | 0.26 | 0.611 |

**Corresponding Author**:
**Xiaohong Chen, M.D.**
Department of Otolaryngology Head and Neck Surgery,
Beijing Tongren Hospital, Capital Medical University
Key Laboratory of Otolaryngology Head and Neck Surgery (Capital Medical University), Ministry of Education
1 Dongjiaominxiang Street, Dongcheng District,
Beijing 100730, P.R. China
Mobile: +86 13911071002
Email: [trchxh@163.com](mailto:trchxh@163.com)
ORCID: [https://orcid.org/0000-0002-3825-2647](https://orcid.org/0000-0002-3825-2647)
