# Supplementary material for: Clinicopathological features and prognostic analysis of 30 patients with laryngeal and hypopharyngeal adenoid cystic carcinoma: a single-center retrospective study
Source: J Cancer Res Clin Oncol. 2026 Apr 8;152(4):84. doi: 10.1007/s00432-026-06449-1 (PMC13062074; doi:10.1007/s00432-026-06449-1)
Supplement: Supplementary file 10 — Supplementary file10. Subgroup mutated genes, nucleotide mutations, amino acid mutations, mutation types, mutation abundance, TMB information [file 432_2026_6449_MOESM10_ESM.zip › Online Resource 10.docx]

| Patient | 1 | 2 | 3 | 4 | 5 | 6 | 7 | 8 | 9 | 10 |
| --- | --- | --- | --- | --- | --- | --- | --- | --- | --- | --- |
| Mutation Gene | CREBBP | MYB-NFIB | MYB-NFIB | KDM6A | CTNNA3-NFIB | MYB-NFIB | CHEK2 | NOTCH | MYB-NFIB | BRCA1 |
| Exon | exon8 | —— | —— | exon7 | —— | —— | exon11 | —— | ­—— | exon10 |
| Nucleotide Mutation | c.1807C>G | —— | —— | c.571C>T | —— | —— | c.1111C>T | —— | —— | c.3971TG |
| Amino Acid Mutation | p.H603D | fusion | fusion | p.Q191X | fusion | fusion | p.H371Y | p.p2514Rfs | —— | p.M1324R |
| Mutation Type | SNV | —— | —— | SNV | —— | —— | SNV | —— | CNV and SV | SNV |
| Mutation Abundance | 20.92% | 50.00% | 26.62% | 29.68% | 32.40% | —— | 88.63% | 60.00% | —— | 5.19% |
| TMB | 0.72 | 2.50 | 0.00 | 0.40 | 10.40 | —— | 1.80 | 3.84 | 0.00 | ­—— |

**Corresponding Author**:
**Xiaohong Chen, M.D.**
Department of Otolaryngology Head and Neck Surgery,
Beijing Tongren Hospital, Capital Medical University
Key Laboratory of Otolaryngology Head and Neck Surgery (Capital Medical University), Ministry of Education
1 Dongjiaominxiang Street, Dongcheng District,
Beijing 100730, P.R. China
Mobile: +86 13911071002
Email: [trchxh@163.com](mailto:trchxh@163.com)
ORCID: [https://orcid.org/0000-0002-3825-2647](https://orcid.org/0000-0002-3825-2647)
